# Social support and smoking abstinence among incarcerated adults in the United States: a longitudinal study

**DOI:** 10.1186/1471-2458-13-859

**Published:** 2013-09-17

**Authors:** Beth Bock, Cheryl E Lopes, Jacob J van den Berg, Mary B Roberts, LAR Stein, Rosemarie A Martin, Stephen A Martin, Jennifer G Clarke

**Affiliations:** 1 Alpert Medical School – Brown University, Centers for Behavioral and Preventive Medicine, The Miriam Hospital, 167 Point Street, Providence, RI 02903, USA; 2 Rhode Island Department of Corrections, 40 Howard Avenue, Cranston, RI 02920, USA; 3 Memorial Hospital of RI, Brown University Center for Primary Care & Prevention, Pawtucket, RI 02860, USA; 4 University of Rhode Island, Social Sciences Research Center, 130 Flagg Road, Kingston, RI 02881, USA; 5 Brown University, Center for Alcohol and Addictions Studies, 121 South Main Street, Providence, RI 02903, USA; 6 Department of Family Medicine and Community Health, University of Massachusetts Medical School, 151 Worcester Road, Barre, MA 01005, USA; 7 Center for Primary Care and Prevention, Memorial Hospital of RI, 111 Brewster Street, Pawtucket, RI 02860, USA; 8 The Miriam Hospital, Centers for Behavioral and Preventive Medicine, Coro West, Suite 309, 164 Summit Avenue, Providence, RI 02906, USA

**Keywords:** Smoking abstinence, Tobacco, Prison, Incarceration, Social support

## Abstract

**Background:**

In the United States, tobacco use among prisoners is nearly three times that of the general population. While many American prisons and jails are now tobacco-free, nearly all inmates return to smoking as soon as they are released back into the community.

**Methods:**

To better understand the role that personal relationships may play in enabling return to smoking, we enrolled former-smokers who were inmates in a tobacco-free prison. Baseline assessments were conducted six weeks prior to inmates’ scheduled release and included measures of smoking prior to incarceration, motivation, confidence and plans for remaining quit after release. We also assessed global social support (ISEL) and a measure of social support specific to quitting smoking (SSQ). Smoking status was assessed three weeks after prison release and included 7-day point-prevalence abstinence validated by urine cotinine, days to first cigarette and smoking rate.

**Results:**

A diverse sample comprised of 35% women, 20% Hispanic, and 29% racial minorities (average age 35.5 years) provided baseline data (n = 247). Over 90% of participants provided follow up data at 3-weeks post-release. Prior to incarceration participants had smoked an average of 21.5 (SD = 11.7) cigarettes per day. Only 29.2% had definite plans to remain smoking-abstinent after release. Approximately half of all participants reported that “most” or “all” of their family (42.2%) and friends (68%) smoked, and 58.8% reported their spouse or romantic partner smoked.

SSQ scores were not significantly predictive of smoking outcomes at three weeks, however, social support from family and friends were each significantly and positively correlated with motivation, confidence, and plans for remaining abstinent (all p values <0.05). These smoking-related attitudinal variables were significantly predictive of smoking outcomes (all p values <0.01). General social support (ISEL) was not associated with smoking-related attitudinal variables or smoking outcomes.

**Conclusions:**

Inmates of smoke-free prisons have a head-start on being smoke-free for life. They have been abstinent well past the duration of nicotine withdrawal and have great financial incentive not to begin smoking again. However, this advantage may be offset by a lack of non-smoking role models among their family and friends, and perceived lack of support for remaining smoke-free.

**Trial registration:**

ClinicalTrials.gov Identifier: NCT01684995

## Background

In the United States (U.S.), tobacco use is the leading cause of preventable morbidity and mortality, contributing to over 400,000 deaths annually [1]. U.S. economic costs due to the negative health consequences of tobacco use are estimated to be close to $200 billion per year [1]. Cigarette smoking in particular is associated with increased risk of heart disease, cancer, chronic pulmonary disease, and stroke [2]. An effective way of reducing the chances of developing such health conditions and avoiding premature death is to quit smoking.

In 2010, over 45 million American adults were smokers, amounting to approximately 19% of the population [3]. Each year, one in eight of these smokers pass through prisons and jails [4,5]. Growing awareness of the harmful health effects of second-hand smoke has prompted many correctional facilities in the U.S. to become tobacco-free. The majority of these prisons (~60%) have complete smoking bans with no tobacco products allowed anywhere in the facility by inmates or staff [6]. In spite of this, virtually all inmates (97%) return to smoking as soon as they are released back into the community [5].

Overrepresented in U.S. correctional facilities are persons who are racial and ethnic minorities, impoverished individuals, and those with mental health and substance use addictions [7-9]. Addressing tobacco use among these highly underserved populations is especially important if we are to reduce health disparities that exist in the U.S. associated with tobacco-related illnesses [10,11]. Despite the great need for smoking abstinence in this population, few studies have examined the factors that promote or inhibit return-to-smoking after release from prison.

To date, no studies have examined the relationship of social influences on maintaining smoking abstinence among individuals after release from a forced-abstinence situation (e.g., prison, hospital inpatients). Most of the literature concerning social relationships and tobacco use comes from studies of individuals who are actively trying to quit smoking [12-15] or observational and epidemiological studies of existing relationships among smokers versus non-smokers [16]. The initiation, maintenance and cessation of smoking are strongly influenced by family members and friends [12-16]. “Buddy” systems and other targeted social support have been used to enhance outcomes in smoking cessation programs [15-22], although the long-term efficacy of interventions designed to increase partner support is equivocal [23]. Partner support, particularly support from a live-in partner or spouse, seems especially important at supporting active cessation efforts and efforts to maintain smoking abstinence [15,23]. The goal of this paper is to examine the influence of personal relationships on the maintenance of smoking abstinence following release from a smoke-free prison.

## Methods

Complete details of the methods for this study as well as the main outcomes of the overall clinical trial have been described elsewhere [24,25]. Methods were approved by the Institutional Review Board and The Office for Human Research Protections prior to initiating recruitment. To further protect study participants, a certificate of confidentiality was obtained.

This study was conducted in a large state correctional facility in the New England region of the U.S. Men and women inmates from a medium security prison who were scheduled for release within the next eight weeks were screened for eligibility. The study research assistants (RAs) interviewed inmates in a private area, explained the nature of the study, and informed individuals that participation was completely voluntary and would not affect any facility privileges or their legal status. Eligible individuals were 18 years of age or older, had smoked 10 or more cigarettes per day prior to incarceration, and were English speaking. The study RAs administered informed consent to all eligible individuals who wanted to enroll. This process included an explanation of the study, consent form read to the individual, questions answered and forms signed. All participants received an American Heart Association smoking cessation pamphlet, a list of community resources and study contact information.

### Measures

After providing consent, each participant completed a 60-minute Audio Computer-Assisted Self-Interview (A-CASI) questionnaire. During A-CASI participants were able to listen to questions on headphones while reading questions on the computer screen, using the computer to input their responses. RAs were present during A-CASI to assist the participant if he or she had questions or experienced technical problems.

Assessments included demographics, smoking history and attitudes, affect and social support. Readiness, Confidence and Motivation to remain tobacco-free following release from prison were each measured with a single item rated on a scale from 1 to 10 (where 1 = “not at all” and 10 = “extremely”). Plans to remain tobacco-free were rated on a scale from 1 to 11 in response to the instruction “Describe how you feel about your smoking now. Think about how much you smoked before you came in to prison and what you plan to do when you get out (choose one)”. The percentage of participants endorsing each response is presented in Table 1. Readiness, confidence, motivation and plans for remaining tobacco-free are referred to collectively as “smoking-related attitudinal variables”. Pack-years smoked was calculated as the number of years since regular smoking began minus the number years of smoking abstinence (e.g., during incarceration or previous quits) times the number of cigarettes (at 20/pack) smoked per day. The nine-item short form of the Temptations to Smoke Inventory (TSI) [26] was used to assess smoking abstinence self-efficacy. Nicotine dependence was also assessed since it has a known impact on the ability of smokers to maintain smoking abstinence following a quit attempt [27-29]. Nicotine dependence was measured by the Fagerstrom Test for Nicotine Dependence (FTND) [30]. The wording of FTND questions was altered to refer to the period of time before incarceration. Possible scores on the FTND ranged from 0–10 with scores of 6–10 indicating high levels of nicotine dependence. The Interpersonal Support Evaluation List (ISEL) [31,32] was used as a measure of perceived general social support across four domains (Appraisal, Belonging, Tangible help, and Self-esteem). The Social Support for Quitting questionnaire (SSQ) contains six items that are averaged to comprise two subscales measuring the influence of family and friends (SSQ_Family and SSQ_Friends, respectively), and include the number of current smokers and successful quitters among the participant’s family and friends, and the participant’s expectations regarding how supportive, or unsupportive these individuals would be if the participant chose to maintain smoking abstinence (Table 2). Three additional items assess smoking in the household and support for remaining tobacco free from a romantic partner.

**Table 1:** Plans for smoking post-release

**Item response**	**Percent (n) of participants endorsing**
I am committed to not smoke when I get out	29.2% (52)
I have begun to make changes (e.g., talked to friends and family) so I don’t smoke when I get out	7.3% (13)
I plan not to smoke when I get out	21.9% (39)
I plan NOT to smoke THAT MUCH when I get out	9.6% (17)
I plan to smoke LESS than I used to when I get out	6.7% (12)
I OFTEN think about not smoking, but I have no plans yet to stay quit when I get out	9.0% (16)
I SOMETIMES think about not smoking, and I have no plans yet to stay quit when I get out	8.4% (15)
I RARELY think about not smoking, I have no plans to stay quit when I get out	1.7% (3)
I RARELY think about not smoking, and I have no plans to stay when I get out	0.6% (1)
I DO NOT think about changing my smoking when I get out	2.8% (7)
I have decided to smoke the same as before or more, when I get out	1.7% (3)

**Table 2:** **Social support for quitting** (**ssq**)

**#**	**Question**	**Scale**	**Percent endorsing**
1	How many of your family members smoke cigarettes?	1-None of them	11.2
		2-Some of them	46.7
		3-Most of them	33.5
		4-All of them	8.7
2	How many of your family members have successfully quit smoking cigarettes?	1-None of them	37.2
		2-Some of them	54.5
		3-Most of them	4.8
		4-All of them	3.5
3	How would your family react to your quitting smoking cigarettes?	1-Will discourage it	0.4
		2-Won’t accept it	0.9
		3- Neutral	14.9
		4-Will accept it	23.8
		5-Will encourage it	60.0
4	How many of your friends smoke cigarettes?	1-None of them	1.3
		2-Some of them	30.8
		3-Most of them	55.6
		4-All of them	12.4
5	How many of your friends have successfully quit smoking cigarettes?	1-None of them	45.5
		2-Some of them	51.3
		3-Most of them	2.2
		4-All of them	0.9
6	How would your friends react to your quitting smoking cigarettes?	1-Will discourage it	3.0
		2-Won’t accept it	0.9
		3- Neutral	21.2
		4-Will accept it	45.5
		5-Will encourage it	29.4
7	How many people living in your household currently smoke?		Mean = 1.83 (SD = 0.96)
8	If married or living together, does your spouse/partner smoke?	1-Yes	58.8
		0-No	14.2
9	If you have a romantic partner, spouse, or boyfriend/girlfriend, how supportive is this person in your efforts to quit smoking?	10 point scale	
		1-Very Unsupportive	
		10-Very Supportive	Mean = 7.3 (SD = 6.9)

Items 1–3 total = Family SSQ; items 4–6 total = Friends SSQ.

Three weeks after release from prison, the study RA contacted participants by phone to conduct follow up assessments including a detailed timeline follow-back (TLFB) [33,34] to assess tobacco use. Individuals who reported abstinence of at least 7 days were asked to come in person to provide a urine sample to test for cotinine. Smoking outcomes at follow up were assessed as (1) 7-day point prevalence abstinence (7PPA), (2) days to first cigarette after release (DFC), and (3) smoking rate as the average number of cigarettes per day (Rate) during the past 7 days.

### Statistical analyses

The purpose of the present investigation was to report on the role of personal relationships and social support on the resumption of smoking after release from a smoke-free prison. First, descriptives are presented for SSQ items including the number of current smokers and successful quitters among the participant’s family and friends, participants’ expectations regarding supportiveness to maintain smoking abstinence, and whether or not participants lived with a smoker. Next, the relationship of the SSQ scales and social support in general (ISEL scales), temptations to smoke, and attitudinal variables is examined with Pearson correlations. Finally, we compared those who remained abstinent and those who smoked at follow-up on whether or not they lived with a smoker and whether or not they had encouragement to remain tobacco-free using chi-square tests. Regression analyses were used to examine the relationship of SSQ scales with smoking outcomes at 3 week follow-up and included, 7-day point-prevalence abstinence (logistic regression), days to first cigarette (cox regression), and smoking rate (multiple regression). The regression models controlled for nicotine dependence (FTND score), gender and depressive symptoms (CES-D) since these variables are related to smoking outcomes [35,36].

## Results

Of the 312 people screened for the study, 273 met eligibility criteria, 262 (96.0%) agreed to participate and completed the consent procedure. Of these, 15 were excluded from data analysis; nine because of a computer error resulting in lost baseline survey data and six because they were never released. Of the remaining 247 participants, 228 (92.3%) completed the three week post-release follow-up assessment. Following intention-to-treat principles, those lost to follow up (n = 19) were counted as having resumed smoking.

### Participant characteristics

Participants (34.7% women) averaged 35.5 years of age (SD = 9.2). Just over half of the sample (51%) identified as non-Hispanic White, 20% identified as Hispanic, and 17.6% identified as Black. The remaining 10.2% identified as other races or mixed-racial background. Over half (64%) had less than 12 years formal education, 20% had graduated high school, and 16% had some education beyond high school. Most participants were single and had no romantic partner (52%), or were not living with that partner (9%), or were living alone due to divorce/separation (7%). One-third were living with a partner while single (25%) or were married (7%).

### Smoking related variables

The average age at which participants began smoking daily was 15.7 years (SD = 4.5). Prior to incarceration, participants had smoked an average of 21.5 (SD = 11.7) cigarettes per day, for an average of 19.2 years (SD = 10.1), with total pack-years averaging 20.6 (SD = 15.9). Nicotine dependence, as measured by the modified FTND, averaged 5.1 (SD = 2.3). 51.1% scored 0–5 (low nicotine dependence) and 48.9% scored 6–10 (high nicotine dependence). Just over half of all participants (51%) planned to resume smoking after release from incarceration (Table 1). The mean score for Plans to remain abstinent was 3.71 (SD = 0.94), being between “3 = I plan not to smoke when I get out” and “4 = I plan not to smoke that much when I get out”. The item measuring plans to remain tobacco-free item was correlated with readiness (r = -.71, p < .01), confidence (r = -.65, p < .01), and motivation (r = -.75, p < .05), but not with participant age or race/ethnicity, FTND score, or number of cigarettes smoked per day before incarceration. Scores on smoking related attitudinal characteristics are presented in Table 3.

**Table 3:** **Smoking**-**related attitudinal characteristics**

	**Mean**	**SD **^**a**^
Remain tobacco-free post release ^b^		
Confidence	5.5	2.8
Readiness	6.6	2.8
Motivation	6.3	2.9
Temptations ^c^		
Habit	10.24	2.7
Negative Affect	12.4	2.5
Social	11.2	2.6
ISEL		
Appraisal	8.6	2.5
Belonging	7.8	2.6
Tangible	8.3	3.0
Self-Esteem	6.4	2.3
Total	31.5	7.7

^a^*SD* Standard deviation.

^b^ Scores range from 1 'not at all’ to 10 'extremely’.

^c^ Smoking abstinence self-efficacy assessed using TSI. Scores range from 2 to 15.

### Smoking-related social influences

The vast majority of participants (88%) reported that at least one of their family members smoked. Nearly half (42.2%) of participants reported that “most” or “all” of their family members were smokers, and over half (68.0%) reported that “most” or “all” of their friends were smokers. Only 11.1% reported having no family members who smoked and virtually none (1.3%) reported having no friends who smoked. Of those who were married or living with a partner before entering the ACI (n = 204), 58.8% said that their partner smoked. Nearly three quarters of participants (67%) indicated that in the three months prior to their incarceration they lived with a smoker. Over a third (37.2%) of participants reported that none or their family members had ever quit smoking, and nearly half (45.5%) reported that none of their friends had ever quit smoking. Despite this, 60% reported that their family would encourage them if they were to decide to quit smoking. However, only 29.4% reported that their friends would encourage efforts to quit (Table 2).

### Social support and smoking variables

The SSQ subscales assessing the potential influence of family (SSQ_Family) and friends (SSQ_Friends) were both positively correlated with the ISEL Appraisal, Belonging and Tangible subscales, but not with the Self-esteem subscale. SSQ_Family was also significantly and negatively correlated with the TSI items measuring smoking temptations in response to social cues and negative affect situations. Both SSQ_Family and SSQ_Friends were significantly and positively correlated with motivation, confidence, and plans to quit (Table 4). None of the ISEL subscales were significantly associated with Motivation, Confidence, or Plans to quit smoking.

**Table 4:** **Correlations between social support**, **smoking temptations**, **and attitudinal variables**

	**SSQ**	
	**Family**	**Friends**
ISEL		
Appraisal	.250**	.288**
Belonging	.264**	.161*
Tangible	.231**	.146*
Self-esteem	.045	.047
Total	.249**	.167*
TSI		
Social	-.134*	-.053
Neg. Affect	-.227**	-.017
Habit	-.128	-.035
Attitude Variables		
Motivation	.186*	.185*
Confidence	.199**	.184*
Plans	.158*	.187*
Readiness	.114	.144

* p < 0.05, ** p < 0.01.

*SSQ* Social Support for Quitting.

*TSI* Temptations to Smoke Inventory.

*ISEL* Interpersonal Support Evaluation List.

### Social support and smoking outcomes

At three week follow up 15.8% of participants were abstinent from smoking (7PPA). Average days to first cigarette was 6.43 (SD = 9.2; range = 0–21), and the average smoking rate at week 3 was 7.56 cigarettes per day (SD = 9.0; range = 0-50).

We compared those who remained smoke-free to those who smoked after release on whether or not they currently lived with a smoker, whether or not a family member or friend had quit smoking, and whether or not family or friends would encourage quitting smoking. Of those who remained abstinent at follow up, 22% lived with no smokers, compared to 12.5% of those who resumed smoking, however this difference was not significant (χ^2^ = 2.4, p = 0.10). Having a family member or friend who had quit smoking was not different between those abstinent (62.2%) and those smoking (62.9%) at follow up. Having family or friends who would encourage a quit attempt was not significantly different between those who were smoking at follow up (59.3% and 37.1% respectively) and those who remained abstinent (63.9% and 28.1%, respectively).

Logistic regression analysis found no significant relationship between SSQ_Family and 7PPA, (top of Table 5) after controlling for FTND, gender, and CES-D, overall model χ^2^ (4, *N* = 212) = 2.17, *p* = .07. Table 5 (Top) displays the unstandardized regression coefficients (*B*), the odds ratio (*OR*), 95% confidence interval (*CI*) and significance value for each predictor. Multiple regression analysis found no significant relationship between SSQ_Family and smoking rate (middle of Table 5) although the overall model *R* was significantly different from zero, *R*^*2*^ = .12, *F*(4, 171) = 5.90, *p* < .01. Table 5 (middle) displays the unstandardized regression coefficients (*B*), the standard error of *B* (*SE*), the standardized regression coefficients (β), *t* and *p* value for each predictor. Cox regression found no significant effect of SSQ_Family on days to first cigarette (bottom of Table 5), nor a significant model with all four predictor variables, χ^*2*^ (4) = 2.57, *p* = .63. Table 5 (bottom) displays the *B*, standard error of *B* (*SE*), hazard ratio (*HR*), 95% *CI*, and *p*-value for each predictor.

**Table 5:** **Regression models for ssq_family predicting smoking abstinence**, **number of cigarettes smoked per day**, **and days to first cigarette at follow**-**up**

Predicting 7PPA					
*Predictor*	*B*	*SE B*	*OR*	*95% CI*	*p*
CESD Score	-.01	.04	.99	[.93 – 1.07]	.92
FTND Score	.05	.08	1.05	[.89 – 1.24]	.53
Gender	-.01	.41	.99	[.45 – 2.23]	.99
SSQ_Family	.48	.38	1.62	[.77 – 3.39]	.20
Predicting Rate					
*Predictor*	*B*	*SE B*	β	*t*	*p*
CESD Score	.06	.12	.04	.47	.64
FTND Score	.64	.29	.16	2.19	.03
Gender	5.95	1.39	.31	4.29	< .001
SSQ_Family	.73	1.25	.04	.58	.56
Predicting DFC					
*Predictor*	*B*	*SE B*	*HR*	*95% CI*	*p*
CESD Score	-.01	.02	.99	[.97 – 1.03]	.93
FTND Score	.01	.04	1.03	[.94 – 1.09]	.74
Gender	.12	.18	1.13	[.79 – 1.62]	.50
SSQ_Family	.22	.16	1.25	[.92 – 1.70]	.15

*Note*. *CI* confidence interval for odds ratio (*OR*). *7PPA* 7 day point prevalence abstinence at week 3 follow up. *DFC* Days to first cigarette – assessed at follow up. *Rate* Average number of cigarettes per day at week 3 follow up.

Regression analyses found no significant relationship between SSQ_Friends and 7PPA (top of Table 6) after controlling for FTND, gender, and CES-D, overall model χ^2^ (4, *N* = 212) = 1.53, *p* = .82. Table 6 (Top) displays the unstandardized regression coefficients (*B*), the odds ratio (*OR*), 95% confidence interval (*CI*) and significance value for each predictor. Multiple regression analysis found no significant relationship between SSQ_Friends and smoking rate (middle of Table 6) although the overall model *R* was significantly different from zero, *R*^*2*^ = .36, *F*(4, 170) = 6.27, *p* < .01. Table 6 (middle) displays the unstandardized regression coefficients (*B*), the standard error of *B* (*SE*), the standardized regression coefficients (β), *t* and *p* value for each predictor. Cox regression found no significant effect of SSQ_Friends on days to first cigarette (bottom of Table 6), nor a significant model with all four predictor variables, χ^*2*^ (4) = 2.68, *p* = .61. Table 6 (bottom) displays the *B*, standard error of *B* (*SE*), hazard ratio (*HR*), 95% *CI*, and *p*-value for each predictor.

**Table 6:** **Regression models for ssq_friends predicting smoking abstinence**, **number of cigarettes smoked per day**, **and days to first cigarette at follow**-**up**

Predicting 7PPA					
*Predictor*	*B*	*SE B*	*OR*	*95% CI*	*p*
CESD Score	-.02	.04	.98	[.91 – 1.05]	.57
FTND Score	.04	.09	1.04	[.88 – 1.23]	.68
Gender	.01	.42	1.01	[.44 – 2.28]	.99
SSQ_ Friends	.44	.44	1.55	[.66 – 3.64]	.31
Predicting Rate					
*Predictor*	*B*	*SE B*	β	*t*	*p*
CESD Score	.09	.12	.06	.79	.43
FTND Score	.58	.29	.14	1.93	.06
Gender	6.22	1.40	.33	4.45	<.01
SSQ_ Friends	.13	1.39	.01	.09	.92
Predicting DFC					
*Predictor*	*B*	*SE B*	*HR*	*95% CI*	*p*
CESD Score	-.01	.02	.99	[.96 – 1.03]	.66
FTND Score	.01	.04	1.01	[.94 – 1.09]	.74
Gender	.19	.18	1.20	[.84 – 1.73]	.32
SSQ_ Friends	.24	.17	1.27	[.91 – 1.77]	.17

*Note*. *CI* confidence interval for odds ratio (*OR*). *7PPA* 7 day point prevalence abstinence at week 3 follow up. *DFC* Days to first cigarette – assessed at follow up. *Rate* Average number of cigarettes per day at week 3 follow up.

Since smoking-related attitudinal variables were related to social support for quitting smoking, exploratory post-hoc analyses were conducted to determine if smoking-related attitudinal variables were also related to smoking abstinence status, days to first cigarette, and smoking rate. Readiness, Motivation, Confidence, and Plans for post-release smoking abstinence were all significantly correlated with smoking outcomes (Table 7; all p values < 0.01). Implications for these post-hoc analyses are discussed below.

**Table 7:** Correlations of attitudinal variables with smoking outcomes

	**Readiness**	**Motivation**	**Confidence**	**Plans**
7PPA	-.30	-.24	-.30	.25
DFC	.49	.44	.49	-.40
Rate	-.43	-.41	-.48	.44

*7PPA* 7 day point prevalence abstinence at week 3 follow up.

*DFC* Days to first cigarette – assessed at follow up.

*Rate* Average number of cigarettes per day at week 3 follow up.

## Discussion

Results of this study demonstrate that former inmates have few social models for non-smoking, and generally lack strong social support from family and particularly from friends relevant to maintaining smoking abstinence after release from prison. Despite prolonged abstinence from smoking, the vast majority of inmates who are former smokers will return to smoking almost immediately upon release from prison [5].

The role of social support in helping people to quit smoking has been the focus of numerous research studies [14,15,35]. While the results of those studies indicate that social support may function in a complex manner, it has been generally found that individuals who attempt to eliminate unhealthy behaviors (e.g., smoking) or to adopt healthy behaviors (e.g., exercising regularly) are more likely to be successful, when they perceive themselves to have higher levels of social support [21].

Results in this study also suggest that temptations to smoke (TSI) appeared more related to family than friends of inmates, but social support for smoking abstinence from both family and friends was significantly related to smoking-related attitudinal measures, including confidence, motivation, and plans for remaining abstinent.

Despite the large body of research on the effects of social support on a range of health behaviors, few studies to date have examined this construct specifically in relation to former inmates. It is known that inmates with higher levels of social support (as determined by factors such as regular visits from friends and family members) generally do better in their daily lives in prison [36]. More supported inmates commit fewer violations of institutional rules and take part in more rehabilitative programs than do inmates with less consistent support [36]. This suggests that inmates who are more likely to follow prison rules and to take part in programs are also more likely to have stronger support networks.

Results obtained in this study suggest however, that global levels of support as measured on the ISEL, while correlated with support for smoking abstinence (SSQ), were not associated with smoking-related attitudinal variables or smoking outcomes. This seems unsurprising given the relative scarcity of non-smoking role models among family and friends of inmates in this study.

For individuals such as recently released former inmates who are trying to remain free of tobacco, a large part of smoking-specific social support involves the proportion of people in one’s social network who smoke cigarettes. The greater the number of people in one’s social network who smoke, the more likely it is the former inmate will be exposed to smoking. Close social bonds in this population appear to provide at least as much encouragement of continued smoking than abstinence. Many study participants had family members who were former smokers, however having a family or friend role-model for quitting smoking may not be particularly pertinent for someone who isn’t engaging in deliberate quitting, but rather, is choosing whether or not to continue abstinence.

The participants in this study, once they were released, returned to environments in which the majority of friends and family members smoke, including almost 60% of those with spouses or live-in partners. Moreover, participants indicated that only a minority of the members of their social networks had experiences with cessation. This indicates a dearth of role models for participants as they attempt to remain abstinent from smoking. However, despite the high rates of smoking among family and friends and the low rates of cessation efforts, more than one half of the participants believed that their family members would encourage participants in their efforts to remain tobacco free. The perception of encouragement from friends was much lower. Less than one-third of the participants believed that friends would encourage them in their efforts to remain abstinent. This difference in participants’ perceptions of support for smoking abstinence support potentially offered by family members and friends suggests that family members may be better able to support them in their efforts to remain smoke-free.

Interestingly, stronger SSQ family support was associated with less social and negative affect temptation to smoke while SSQ friends support was not associated with smoking temptation. However, the ISEL’s general measure of social support was not associated with smoking in response to negative affect or habitual smoking. Hence, it may be that only specific support from family for smoking abstinence is associated with less temptation to smoke. This suggests that interventions that include family may be more effective in helping former inmates remain smoke-free after release than interventions targeted only to the individual inmate.

**Figure 1:**
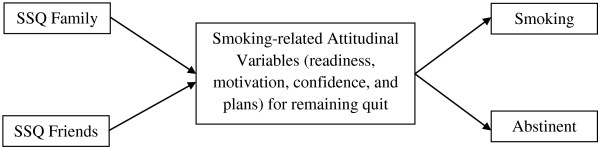
Proposed pathway of SSQ variables to smoking-related attitudinal variables and smoking outcomes.

While there was no direct association between perceived social support for smoking abstinence from either family or friends and smoking outcomes, the relationship of SSQ variables to the smoking-related attitudinal variables of motivation, confidence, and plans for remaining quit, and the relationship of these variables to smoking outcomes suggests a possible pathway (Figure 1) that merits further exploration in future research. Confidence was significantly and negatively correlated with potential negative influence of family while both Motivation and Plans for remaining quit were significantly correlated with influence of both family and friends.

## Conclusions

Individuals who have been incarcerated in a smoke-free prison are in an excellent position to remain smoke-free upon release. However, they face significant barriers including a lack of non-smoking role models among family and friends, and variable levels of abstinence-specific social support from family and friends. Interventions are needed for this population which include both the individual and his/her family or social network.

## Competing interests

The authors declare that they have no competing interests.

## Authors’ contributions

JC, BB, LS and RM developed the study design. MR and RM organized the data and conducted the statistical analyses. BB, JV, and CL were the primary writers of the manuscript and all authors were involved in editing the manuscript. All authors read and approved the final manuscript.

## Pre-publication history

The pre-publication history for this paper can be accessed here:

http://www.biomedcentral.com/1471-2458/13/859/prepub
